# Local Injuries Justifying the Amputation of a Limb

**Published:** 1865-01

**Authors:** Frank A. Ramsay

**Affiliations:** St. Bartholomew's; Surgeon and Medical Director P. A. C. S.


ART. II.—"*Local Injuries Justifying the Amputation of a Limb.*" By Prof. SKEY, St. Bartholomew's, abridged for publication by FRANK A. RAMSAY, Surgeon and Medical Director P. A. C. S.

Where is the standard of surgery by which we are guided in the amputation of limbs? In truth, there exists no standard; and the consequence is, that the limb which is preserved in one hospital or in one locality, is amputated in another. Now, this is a grievance which may be fairly urged against the surgical profession by that large community for whose benefit we profess to have ascertained and adopted the most eligible means of curing diseases and of relieving the consequences of local injuries. It is doubtless a question of difficulty, which can only obtain a solution through the medium of experience. But the means of obtaining this experience are denied to the individual, and can only be obtained in the aggregate. And hence the inconsistency of professional practice, the cause of which forms a very legitimate subject for examination and inquiry.

It would appear to arise from various causes operating upon the mind, and hence determining the conduct of the operator. The first, and perhaps the most impressive in its influence, consists in the different degree of reliance placed by different surgeons on the power and resources of Nature in the cure of diseases. Men adopt different views of the power of the curative art. With some it holds the relation of a *vicegerent;* with others, of a *handmaid.* Our minds are not universally impressed by the conviction that Nature cures diseases, and not we; and that the province of the surgeon, beyond which he cannot step one foot, consists in removing obstacles in her path. If this wholesome fact was impressed more deeply on the professional mind, would it not instinctively lead to a closer observation, and, of necessity, to a higher appreciation of her powers. At a period as late as half a century back, amputations were at least three times more frequent than at present. Why are they now less frequent? Not because severe injuries are wanting, or that diseases have proved universally tractable, but because by the study of physiology we have become, comparatively speaking, familiar with the power which Nature wields, and by the observation of the greater resources of the body than were known to our grandfathers.

Secondly, because I do honestly believe that there exists a higher tone of feeling in the professional mind, a higher appreciation of the value of human life, and a truer sympathy with human suffering. I believe, also, that a very general observance will corroborate my own impression that, as a rule, the greater the experience of the surgeon, the fewer the operations, and, as a necessary inference, his greater reliance upon Nature. I am quite convinced that these powers have yet to be fully ascertained; and, when tested by the few, they will eventually be acknowledged by the many. This progress of medical opinion is elaborate and slow, but it is not the less sure.

Thirdly, because we have identified our opinions with what I believe to be most erroneous views, propounded by our forefathers on the subject of what were termed secondary operations. The theory of this almost universal law of surgical practice is this: That it is better for the constitution to bear one shock than two; and no reflecting man will deny it. It therefore resolves itself into the necessity of an immediate decision, "Now or never." "Now" saves life, but at the expense of a limb; "Never" risks life, and it is our first duty to preserve it. But this well-sounding theory, although it proclaims truth in the abstract, does not proclaim truth in the application. It is true to the letter, not to the spirit. The exceptions to its application are infinite; strictly speaking, it only applies in full force to cases of extreme injury, such as large contusions, crushings, or lacerations of the limb, recovery from which is beyond hope or appeal. Consequent on such destruction, the constitution must necessarily sink; and the earlier the amputation the more probable the recovery, because collapse of the vital powers is the certain issue, and reaction is impossible.

And here let me ask a question not inappropriate to the purpose. After what period of time, from that of the injury, may an operation be undertaken, which brings it within the category of the laws? At the expiration of how many hours is a primary converted into a secondary operation? By *immediate amputation,* I understand the removal of a shattered limb, as practised on the field-of-battle, when the sufferer is removed to the rear for that purpose. But, if several hours elapse, do we still retain the term "immediate amputation." We make no distinction between an interval of one hour and a quarter of a day, on which distinction everything depends. If, then, we lose by distant absence from the case, (a very frequent condition of things,) the advantage of immediate amputation is that not an additional element in favor of the treatment which appeals to Nature, and which calls upon her to put forth her powers of restoration. Mr. Abernethy used to dwell with much force upon the curative powers of the constitution in cases of compound dislocation of the foot at the ankle-joint. He related three examples of this injury, which he treated successfully, after condemnation to the knife by other eminent surgeons. Had Mr. Abernethy's experience been greater at that period of his life, he would not have made these the only exceptions to a rule that requires far larger limitations.

It would be well for the cause, both of humanity and of science, if it were possible to draw a definite line, which should determine the confines of reparative power; but it is the very impossibility of doing so which appears in some measure to justify so wide a range in the practice of different surgeons. That there is a line, obscure to our senses though it be, will hardly be disputed by any one who has deemed the fact worthy of remark—how common is the resort to the knife in some districts compared with that of others. And the difficulty of decision is unquestionable, such is the complication of the requisite injury. Notwithstanding which, some approach may be attempted to a more general rule, founded on the nature and extent of the injury, than has hitherto guided the hospital surgeon in his decision, although the question of local injury will be subject to large modifications, founded on that of age, sex, the character and condition of constitution of the affected person.

These examples may be divided into—first, such as *require* amputation; secondly, such as *justify* amputation; and thirdly, such as neither *require* nor *justify* amputation. One word on the definition of the term *justify* amputation. To justify may be not to give the reality, but the appearance only, of justice; to obtain the sanction of the world. A man may obtain the warrant of general opinion, while he fails to possess his own. He may be safe from comment or criticism, while he is amenable to denunciation *in foro conscientiæ.* By the term "justify," I mean that warrant in favor of the removal of a limb which is obtained from the consideration of an injury placed on the confines of necessity, especially if occurring in early life, in advanced age, or in impaired constitution.

Of the structures entering into the composition of a limb, one and all of which are the subject of rupture or disorganization, the first importance perhaps justly attaches to the arterial system; but as universal experience teaches us that the channel of the main artery may be suddenly obliterated without danger to the vitality of the limb, so in the rupture of the main arterial trunk, evidenced by the pulseless condition of the limb below, the injury is, *per se*, no warrant for amputation. But if, superadded to the rupture of the main artery, the muscular system at the seat of injury is largely contused or ruptured, and the collateral channels for arterial, as well as venous blood are involved in the injury, it is more than probable that the limb will quickly fail in nourishment, the indication of which is obtained from the loss of temperature. This loss, if complete, will become apparent in the course of an hour or two. But the loss is rarely complete, and several hours, or even a day, may be required to determine the affirmative of the mischief done, or this evidence. But it is all-important evidence, and fully justifies the postponement of the decision.

Next in importance to arteries, and in close relation to them, stands the nervous system; and in reference to injury to the chief nerve or nerves, the same remark will almost apply. We do not amputate the leg because the sciatic nerve is rent asunder. The true principles of surgery would dictate a pause—a period for watching and observation. But as the rupture, whether of artery or nerve, is commonly a matter of uncertainty, we can only judge by consequences, and for these we should wait.

To justify amputation, from rupture or laceration of the muscular system of a limb, the injury must be very great, because the constitution does not sustain a shock in proportion to the extent of the injured surface, supposing the integuments to remain unbroken; but if the muscles be largely torn, and the investing integuments detached, and not susceptible of entire, or nearly entire replacement, I confess such an injury would justify a doubt as to the power of Nature to restore the parts to health. I speak of a very large laceration, with contusion of muscle, coupled with separation of integument and extravasation of blood.

I do not concur with many surgeons, who deem exposure of the cavity of a joint an important element of failure. I am quite aware that it is so generally deemed, and I recall to my recollection the early part of my own professional life, when a compound dislocation of a joint alone was deemed a warrant for the amputation of a limb. But I can bear witness to many cases of recovery as regards the limb, and a few of recovery to the joint itself.

Undue importance appears to me to be attached also to fracture into a joint, as though such fracture, in reference to the retention of a useful limb, raised a serious obstacle to recovery. That it places the joint in jeopardy I readily allow, but I do not believe that the advocate for amputation in any given case can derive from its presence an argument of great force, although I do not deny that it should always be considered an aggravation of the mischief done. The same remarks will apply to fracture of the bone, especially if comminuted, when superadded to the larger injury of a rupture of the main artery, or of the main nerve, extensive rupture of muscles, or laceration and disorganization of the integuments.

I have never yet observed much advantage to accrue to the patient from the introduction of the finger, through an opening in the skin, which is employed as an explorer, and carried round in all directions, for the purpose of ascertaining the nature and extent of the injury done. I have never, myself, acquired much knowledge by this process, which could be rendered available to the service of the patient, nor have I known it to be obtained by others. To the patient himself, so far as he is entitled to an opinion, it has always appeared to be positively objectionable. To be sure, it gratifies curiosity, though at some expense of suffering.

Finally, in all doubtful cases, I would give the benefit of the doubt to the patient, and endeavor to restore the limb. If, consequent on a large injury to the leg or thigh, upper arm or forearm, the foot or hand lose their natural warmth, amputation is the only resource. If we find extensive laceration of muscles, with extensive separation of integument, and especially if the integument be disorganized and insusceptible of replacement, I fear we must amputate, even without waiting for the above evidence of loss of vitality in the extremity; but, in a subject moderately healthy, I do not consider that any degree of comminution of bone, or laceration of muscles, unless very extensive; any fracture in a joint, or compound dislocation of a joint, can justify the abandonment of the case, so long as the structures are capable of some general replacement, and the patient can submit without suffering to the restraint necessary to his recovery.

